# Multicenter Study of the Effectiveness of Antifungal Stewardship Team Intervention for Candidemia in Japan in 2008–2021

**DOI:** 10.3390/idr16020027

**Published:** 2024-04-15

**Authors:** Mieko Tokano, Norihito Tarumoto, Jun Sakai, Kazuo Imai, Sakaru Koizumi, Haruka Karaushi, Tamotsu Hatanaka, Etsuko Kishi, Masafumi Seki, Koutaro Mitsutake, Shigefumi Maesaki

**Affiliations:** 1 Department of Infectious Disease and Infection Control, Saitama Medical University, 38 Morohongo, Moroyama, Saitama 350-0495, Japan; mtokano@saitama-med.ac.jp (M.T.);; 2 Departments of Allergy and Immunology, Faculty of Medicine, Saitama Medical University, 38 Morohongo, Moroyama, Saitama 350-0495, Japan; 3 Department of Infection Control, Saitama Medical University Hospital, 38 Morohongo, Moroyama, Saitama 350-0495, Japan; 4 Division of Pharmacy, Saitama Medical University International Medical Center, Hidaka, Saitama 350-1298, Japan; 5 Department of Infectious Diseases and Infection Control, Saitama Medical University International Medical Center, Hidaka, Saitama 350-1298, Japan

**Keywords:** antifungal stewardship team (AFT), candidemia, action bundle, endophthalmitis, ophthalmology

## Abstract

Candidemia, linked to high mortality rates, requires prompt antifungal therapy for better outcomes. Treatment is structured as an action bundle, which is beneficial when followed closely. However, the Japanese action bundle lacks detailed guidance on severe complications like endocarditis or ocular issues. To address this, we adjusted the action bundle and assessed outcomes with and without AFT intervention. We strengthened protocols for blood cultures and organ assessments, and the AFT contacted the primary physician when yeast-like fungi were detected in the patient’s blood culture bottles. Analyzing 204 candidemia cases from 2008–2021, we observed increased adherence and reduced mortality post-AFT intervention. Ophthalmology consultations rose significantly, but many patients had only one visit, suggesting inadequate follow-up. If endophthalmitis is diagnosed, a change in the treatment approach may be necessary. There is a possibility that abnormal ocular findings will be detected during subsequent visits, which highlights the need for improvement in ophthalmology follow-up rates as a future challenge for our AFT activities.

## 1. Introduction

The incidence of invasive fungal infections (IFIs) has significantly increased in the last few decades. In particular, candidemia constitutes a major component of healthcare-associated IFIs [1]. The most common species of *Candida* in Japan are *Candida albicans*, *Candida parapsilosis*, *Candida glabrata*, *Candida tropicalis,* and *Candida krusei* [2]. *Candida* species are endemic to the human skin, gastrointestinal tract, and vagina [3]. *C. albicans*, in particular, has a high distribution frequency in the gastrointestinal tract. *C. parapsilosis* is more likely to be endemic on the skin and is a cause of catheter-associated bloodstream infections [4]. The risk of candidemia has been found to be increased in immunocompromised populations, including critically ill patients, patients on immunosuppressive drugs, neutropenic patients, elderly individuals, patients with *Candida* colonization, and postoperative patients [5]. The use of central venous catheters and the administration of broad-spectrum antibiotics are also risk factors [6,7,8,9]. Candidemia is associated with high mortality rates of 30–50% [10]. In addition, ocular candidiasis, including endophthalmitis, is reported as a serious complication in patients with candidemia [11]. Therefore, the treatment to be given for candidemia has been presented as an action bundle [12,13], and compliance with the action bundle is expected to improve the prognosis [14]. Follow-up by an Antifungal Stewardship Team (AFT) is essential for the provision of appropriate antifungal treatment based on the action bundle. In Japan, the Antimicrobial Stewardship Team (AST) and AFT have intervened in cases involving patients with bacteremia or fungemia in many hospitals since the establishment of additional support for the appropriate use of antimicrobial agents in 2018. The action bundle recommended in Japan [12] includes the following items: (1) removal of existing central venous catheters within 24 h of diagnosis, (2) initial appropriate selection of antifungals, (3) initial appropriate dosing of antifungals, (4) ophthalmological examinations, (5) follow-up blood cultures until clearance of candidemia, (6) assessment of clinical efficacy on the third to fifth day to consider the necessity of alternative therapy, (7) appropriate choice of alternative antifungals, (8) at least two weeks of therapy after documented clearance of *Candida* from the bloodstream and resolution of attributable symptoms (prolonged therapy for candidemia with organ involvement), and (9) step-down oral therapy for patients with a favorable clinical course.

However, some of these items may not apply to all patients (e.g., patients without inserted devices). Therefore, instead of evaluating the entire bundle as a whole, it is necessary to assess compliance with each individual item included in the bundle. Furthermore, there are no detailed descriptions regarding the evaluation and examination of severe complications of candidemia, such as infective endocarditis or intraocular inflammation.

Therefore, we adjusted the action bundle and investigated whether there were differences in the treatment outcomes of candidemia managed with and without AFT intervention.

## 2. Materials and Methods

### 2.1. Patients and Fungal Isolates

All patients with *Candida* spp. detected in blood samples from Saitama Medical University Hospital and Saitama Medical University International Medical Center—a 1000-bed and a 700-bed tertiary care hospital and referral center, respectively—in Saitama, Japan, from 2008 to 2021 were included in this study. Two hundred four patients were finally enrolled. All isolates derived from blood cultures since 2014 were identified by a MALDI Biotyper with the MALDI Biotyper 3.1 software program and MALDI Biotyper Reference Library version 4.0.0.1 (Bruker Daltonics, Bremen, Germany) according to the manufacturer’s instructions, using an autoflex speed mass spectrometer (Bruker Daltonics) [15]. All isolates derived from blood cultures prior to 2014 were identified by CHROMagar^®^Candida (Kanto Chemical Co., Tokyo, Japan). Antimicrobial susceptibility testing was performed by Microscan Walk Away 96 Plus (Beckman Coulter, Brea, CA, USA).

### 2.2. Antimicrobial Stewardship Programs

Since 2018, the AFT has been involved in the treatment of patients with candidemia. The members of the AFT include infectious disease physicians, pharmacists, medical biologists, and nurses. The AFT contacted the primary physician when yeast-like fungi were detected in the patient’s blood culture bottles. We adjusted the previous action bundle [12] by strengthening the collection of two sets of blood cultures and scrutinizing organ involvement (performing echocardiography to examine for infective endocarditis when blood cultures remained persistently positive and conducting an examination by an ophthalmologist, including follow-up in all cases).The AFT provided the following advice to attending physicians: (1) collection of two sets of blood cultures, (2) removal of intravital devices if applicable, (3) use of appropriate antifungal medications (drug type, dosage, length of treatment, assessment of clinical efficacy, make appropriate changes to alternative antifungal therapy), (4) examination by an ophthalmologist including follow-up, (5) follow-up of blood cultures until clearance of candidemia, and (6) scrutiny of organ involvement (e.g., infective endocarditis) when blood cultures are consecutively positive.

In cases where complications of endophthalmitis were suspected, the AFT recommended treatment with fosfluconazole or liposomal amphotericin B. The duration of antifungal drug administration was set to be at least two weeks after the blood culture turned negative. In cases of disseminated candidiasis, the duration of treatment was extended based on the patient’s condition. For example, in cases of concomitant endophthalmitis, a treatment duration of at least three weeks was recommended [12].

### 2.3. Clinical Parameters

The patients were grouped according to the onset period of the candidemia into a pre-intervention group (2008–2017) and a post-intervention group (2018–2021). We collected patient information retrospectively from the electronic medical records of the hospitals. The following data were collected: age, sex, patient risk factors (underlying disease, use of immunosuppressants, steroids, and anticancer drugs, hemodialysis, and neutropenia—neutrophil count <500 cells/m^3^), source of infection (which was reviewed retrospectively and described as “unknown” if not described in the medical records or determined by the attending physicians), use of urinary catheters and central venous catheter, administration of high-calorie infusions, antifungal therapy, sensitivity, retesting of blood cultures, extraction of artifacts, ophthalmology consultation, search for infective endocarditis (e.g., echocardiography), number of sets of blood culture bottles submitted, and 30-day mortality (defined as death due to candidemia). Appropriate antifungal therapy was defined as the use of an antimicrobial agent to which isolates were susceptible based on in vitro susceptibility testing. Although the etiology of candidemia varies among patients, antifungal therapy was determined to be appropriate if the proper treatment duration was adhered to for each patient. The dosage of antifungal was deemed appropriate if it adhered to the recommended dosage by the Japanese guidelines [12]. For micafungin, a dosage of 100 mg or more was considered an appropriate administration. Clinical efficacy was assessed on the third to fifth day. If the effectiveness was insufficient, the necessity of alternative therapy was considered, taking into account sensitivity and tissue penetration. If the condition stabilized and oral medication became feasible, step-down therapy was suggested, but this aspect was not included in the analysis in this study.

### 2.4. Statistical Analysis

Count data were expressed as percentages, and univariate analysis was conducted using Fisher’s exact test. Unpaired Student’s *t*-tests were used to analyze normally distributed measurement data. The Wilcoxon rank-sum test was used to analyze nonnormally distributed measurement data. A logistic regression analysis was performed to analyze the relationship between compliance with the action bundle and the 30-day mortality rate. The log-rank test was used to compare the two survival curves. All statistical analyses were performed using the EZR software program version 1.55 (Saitama, Japan). *p* values of <0.05 were considered statistically significant.

## 3. Results

### 3.1. Patient Background and Candida Species

The patient background information is presented in the upper part of Table 1A. Candidemia was more frequent in the post-intervention group than in the pre-intervention group. The time taken to obtain blood culture results was 3.3 days. The most common underlying diseases in the patients were gastrointestinal diseases (40.2%) and diabetes mellitus (26.5%). A total of 31.7% of the patients were receiving steroids or immunosuppressive drugs, 45.6% required full assistance with activities of daily living (ADLs), and 78.9% had central venous catheters. Significantly more patients in the post-intervention group required full assistance with ADLs. There was no significant difference detected between the two groups in terms of patients’ underlying diseases. In addition, there was a higher incidence of catheter-related infection (CRBSI) in the post-intervention group. In the late post-intervention group, the rates of *C. glabrata* and *C. tropicalis* were increased, while the rate of *C. albicans* decreased in comparison to the pre-intervention group (Table 1B, there were no significant differences in either group). There were no significant changes in susceptibility to antifungal drugs in either group.

### 3.2. Antifungal Drugs Received by Patients

**Figure 1:**
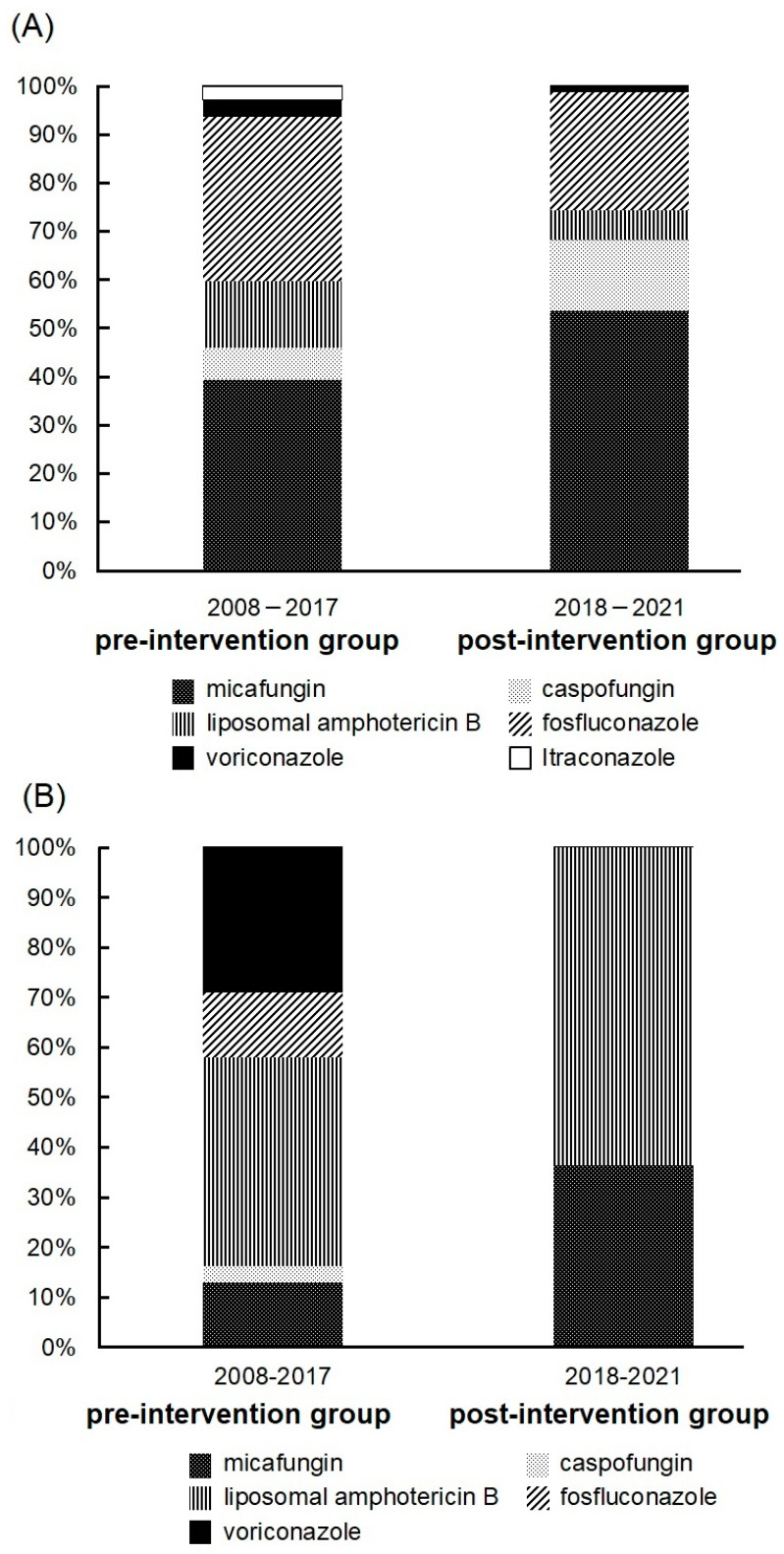
(**A**) Initial antifungal drugs received by patients. The pre-intervention group (2008–2017) and the post-intervention group (2018–2021) included 120 and 84 patients, respectively. (**B**) The drug of choice in cases where the drug was changed due to inadequate efficacy. Drug changes due to diagnosis of endophthalmitis, sensitivity, identification of bacteria, and drug changes due to mild disease were excluded. The pre-intervention group (2008–2017) and the post-intervention group (2018–2021) included 31 and 11 patients, respectively.

The initial antifungal drugs received by the patients are shown in Figure 1A. In both groups, the most commonly used initial antifungal drugs were echinocandins, and there was a rising trend in their use. The proportion of patients receiving >100 mg of micafungin increased in the post-intervention group (34 patients, 77.3%) in comparison to the pre-intervention group (28 patients, 65.1%), although the difference was not statistically significant. Furthermore, there was no difference in mortality between the group treated with >100 mg of micafungin and the group treated with ≤100 mg of micafungin (data not shown). Next, we examined cases in which the drug was changed due to inadequate efficacy. Drug changes due to the diagnosis of endophthalmitis, sensitivity, identification of *Candida* spp., and drug changes due to mild disease (step-down therapy) were excluded. The average time to change the drug was 12 days. The most frequently selected drug was liposomal amphotericin B (Figure 1B). In addition, the group that received echinocandins and the group that received nonechinocandin drugs were compared. Echinocandins were commonly used in cases with renal impairment at the initiation of medication (Table 2). There was no difference in mortality between the two groups.

### 3.3. Effects of AFT Intervention

**Figure 2:**
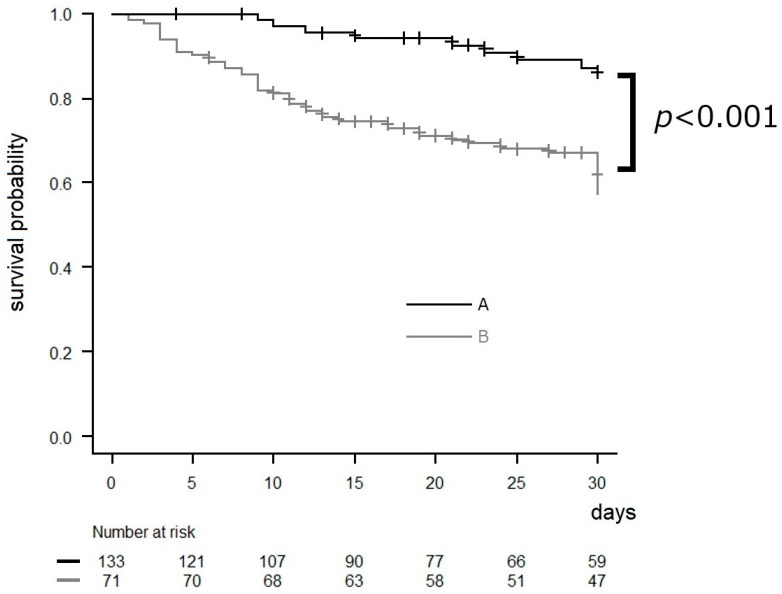
Kaplan–Meier survival curves of survival data in relation to three bundle items (collection of two sets of blood cultures, consulting an ophthalmologist, and follow-up blood cultures until clearance of candidemia), with a follow-up period of 30 days from the date on which the blood culture was submitted (total n = 204). Some patients may not have certain bundle components applied (e.g., patients without inserted devices), so we mainly analyzed three items applicable to all patients. Censored values (+) indicate the last known follow-up time for those subjects still alive after a diagnosis of candidemia. A: patients who had all three items, B: patients who had either zero, one, or two out of the three items among the bundle. *p* value was determined by a log-rank test.

Since 2018, the AFT has been involved in the treatment of patients with candidemia. As a result of AFT intervention, the post-intervention group that received AFT intervention showed improved compliance with the action bundle in all categories in comparison to the pre-intervention group (lower part of Table 1A). Cases where infective endocarditis was detected by echocardiography were observed in both groups, with one case each. All patients were alive at the 30-day mark. In the pre-intervention and post-intervention groups, four patients (3.3%) and seven patients (8.3%), respectively, were diagnosed with endophthalmitis. Out of the 11 patients diagnosed with endophthalmitis, only two reported experiencing subjective symptoms. Out of the nine individuals who did not complain of ocular symptoms, four had impaired consciousness. Another patient was affected by cataracts and was unable to communicate their symptoms accurately. The period between a positive blood culture and the diagnosis of endophthalmitis ranged from zero to nine days, with a median duration of one day. Among the 11 patients with endophthalmitis, one patient was diagnosed with endophthalmitis during their second visit to the ophthalmologist. Two patients received intravitreal injections. AFT intervention led to improved compliance with the action bundle and a decreasing trend in mortality. Next, we analyzed the relationship between items in the action bundle and mortality. Some patients may not have certain bundle components applied (e.g., patients without inserted devices), so we mainly analyzed three items applicable to all patients. Among the items applicable to all patients, “Appropriate antifungal therapy” was adhered to by most physicians, with a relatively high compliance rate. Therefore, we conducted a multivariate analysis on the three bundle items other than “Appropriate antifungal therapy” among the items applicable to all patients: collection of two sets of blood cultures, consulting an ophthalmologist, and follow-up blood cultures until clearance of candidemia. The results indicated that blood culture retesting was associated with lower mortality (Table 3), and patients who had all three items were significantly more likely to survive for 30 days in comparison to those who had either zero, one, or two out of the three items (Figure 2).

## 4. Discussion

Candidemia is associated with a high mortality rate, and it is well known that the delayed initiation of appropriate antifungal therapy leads to poorer clinical outcomes [16]. Strategies aimed at improving adherence to guideline recommendations, such as the optimal management of antifungal agents and timely initiation of treatment for candidemia, have proven to be effective in reducing mortality rates. In particular, it has been suggested that follow-up by an AFT and the implementation of a bundle of care can improve patient outcomes [16]. We investigated a total of 204 cases managed from 2008 to 2021. The proportion of fungal species causing candidemia remained similar to previous reports [2,17], with an increase in non-albicans *Candida*. The susceptibility to antifungal drugs and the detection rate of *Candida* did not show significant changes. The widespread implementation of guidelines resulted in an increase in the use of echinocandins, particularly in severe cases with renal impairment. This was likely because echinocandins do not depend on renal function. Despite AFT intervention, the change in the duration until medication in cases of treatment failure was longer than the recommended period (three to five days) [12]. It was suggested that earlier intervention for medication change is necessary in the future.

In our study, despite the higher proportion of severely ill patients requiring full assistance with ADLs in the post-intervention group, we observed a decrease in mortality rates through active intervention by the AFT from 2018. The proportion of obtaining two sets of blood cultures increased, but no association was observed with a decrease in mortality. This is thought to be because the practice of obtaining two sets of blood cultures had already been established before AFT intervention in cases of bloodstream infections. Repeat blood culture was found to be associated with a decrease in mortality rates. It is believed that AFT was able to guide the attending physician to the appropriate duration of treatment by confirming the negative results of blood cultures. Compliance with the action bundle after AFT intervention showed an increase in all items in comparison to pre-AFT intervention. However, the rate of increased removal of intravital devices and the rate of patients receiving follow-up visits by ophthalmologists were lower in comparison to other items in the action bundle. The removal of intravital devices was performed not only for bloodstream infections caused by *Candida* but also for infections caused by pathogens other than *Candida*, suggesting a preexisting practice.

Ocular candidiasis is one of the major complications in patients with candidemia. It is associated with the risk of developing severe sight-threatening *Candida* endophthalmitis [18]. *Candida* endophthalmitis is rare in patients with candidemia, but in one report, ocular candidiasis was observed in 15.2–26.5% of candidemia patients [11,19,20]. A previous study reported that the probability of diagnosing ocular candidiasis in patients with candidemia within seven days of a positive blood culture was approximately 80.0% [20]. Therefore, ongoing follow-up by an ophthalmologist is crucial both at the time of the diagnosis of candidemia and afterward. In our study, 10 of the 11 patients with endophthalmitis were diagnosed within seven days, while the remaining patient was diagnosed during their second visit to the ophthalmologist, nine days after the diagnosis of candidemia. Our study revealed that many cases concluded with a single visit for the diagnosis, and the rate of subsequent ophthalmology visits was low. There is a possibility of detecting ocular involvement during follow-up visits, and increasing the rate of ophthalmology revisits is a future challenge for our AFT. In contrast, a recent systematic review found that the rate of endophthalmitis from candidemia in routinely screened patients was <1%, and the necessity of a routine ophthalmology consultation in patients with candidemia has consequently been challenged [21]. In our study, the post-intervention group, for which routine ophthalmology visits were recommended, had a higher prevalence of endophthalmitis in comparison to the pre-intervention group. This suggests the possibility of missed cases of endophthalmitis in the pre-intervention group. Among patients diagnosed with candidemia, the number of patients diagnosed with endophthalmitis was low, and there may be cases where a lesion that is presumed to be *Candida* is not actually infectious. However, the most commonly used echinocandins in candidemia have poor ocular penetration and are not appropriate for the treatment of endophthalmitis. Therefore, if endophthalmitis is diagnosed, a change in the treatment approach may be necessary. In addition, in cases of candidemia accompanied by endophthalmitis, a longer duration of treatment is required in comparison to cases without endophthalmitis [22,23]. Furthermore, many severely ill patients with candidemia experience impaired consciousness and cognitive decline, which makes it difficult for them to self-report visual symptoms. Relying on patient-reported symptoms alone may lead to delays in the diagnosis and treatment. In our study, a significant number of patients who were unable to report subjective symptoms had impaired consciousness. Therefore, routine ophthalmic examination is considered important.

The present study was associated with several limitations. First, this was a retrospective study with a relatively small sample size that was conducted at two facilities, which may have introduced selection biases. Second, severely ill patients with candidemia may have died before receiving an ophthalmic examination or undergoing repeat blood culturing, potentially resulting in an inability to implement the action bundle. Third, among the study limitations, it should be added that a severity index assessment (i.e., APACHE II, SOFA) was not included, thus limiting the ability to address mortality. Additionally, given the variability in patient severity, there is a possibility of cases with poor outcomes due to the severity of underlying conditions despite appropriate management of candidemia. Furthermore, there are missing sensitivity data for some cases before 2013.

## 5. Conclusions

In our study, as in previous reports, adherence to the bundle was associated with improved outcomes in candidemia. However, many cases ended with only one visit, indicating a low rate of follow-up visits, and inadequate follow-up regarding endophthalmitis was observed in some cases. There is a possibility that abnormal ocular findings will be detected during subsequent visits, which highlights the need for improvement in ophthalmology follow-up rates as a future challenge for our AFT activities.

## Figures and Tables

**Table 1:** (**A**) Characteristics of patients and compliance with the action bundle. (**B**) *Candida* species isolated from blood cultures.

(**A**)								
		**Total = 204**		**Pre-Intervention Group = 120**		**Post-Intervention Group = 84**		***p* Value**
		**n**	**%**	**n**	**%**	**n**	**%**	
Characteristics of patients								
	Age, median, years	72		74		71		0.82 ‡
	Male sex	133	65.2	79	65.8	54	64.3	0.88 †
	Gastrointestinal disease	82	40.2	51	42.5	31	36.9	0.47 †
	Diabetes mellitus	54	26.5	27	22.5	27	32.1	0.15 †
	skin disease	31	15.2	23	19.2	8	9.5	0.07 †
	Blood disease	27	13.2	18	15.0	9	10.7	0.41 †
	Urological disease	20	9.8	13	10.8	7	8.3	0.64 †
	Collagen disease	10	4.9	5	4.2	5	6.0	0.74 †
	Febrile neutropenia	12	5.9	8	6.7	4	4.8	0.77 †
	Receiving steroids or immunosuppressive drugs	65	31.9	38	31.7	27	32.1	1.00 †
	Anticancer drug	41	20.1	24	20.0	17	20.2	1.00 †
	Radiation therapy	13	6.4	7	5.8	6	7.1	0.78 †
	Required full assistance with ADLs	93	45.6	44	36.7	49	58.3	<0.01 †
	Use of central venous catheter	161	78.9	94	78.3	67	79.8	0.86 †
	CRBSI	89	43.6	43	35.8	46	54.8	<0.01 †
	Use of urinary catheters	162	79.4	95	79.2	67	79.8	1.00 †
	Intravital devices	158	77.5	95	79.2	63	75.0	0.50 †
	Administration of high-calorie infusions	144	70.6	88	73.3	56	66.7	0.35 †
	Received surgery	96	47.1	57	47.5	39	46.4	0.89 †
Number of days until blood culture results are known		3.3		3.4		3.2		0.42 ‡
Patients who did not receive antifungal drugs		13	6.4	11	9.2	2	2.4	0.08 †
The average time to change the drug due to inadequate efficacy		12		12.9		9.4		0.25 ‡
Frequency of candidemia		0.15		0.15		0.16		0.57 †
(number of positive sets/numbers of total sets*100)								
Candidemia patients per 1000 new admissions		0.47		0.4		0.62		<0.01 †
Compliance with the action bundle								
	Collection of two sets of blood cultures	121	59.3	55	45.8	66	78.6	<0.01 †
	Removing intravital devices	124	66.7 (total = 186)	66	59.5 (total = 111)	58	77.3 (total = 75)	0.01 †
	Appropriate antifungal therapy	179	87.7	105	87.5	74	88.1	1.00 †
	Consulting an ophthalmologist	106	52.0	42	35.0	64	76.2	<0.01 †
	Ophthalmology re-examination	37	34.9 (total = 106)	11	26.2 (total = 42)	26	40.6 (total = 64)	0.15 †
	Follow-up blood cultures until clearance of candidemia	147	72.1	68	56.7	79	94.0	<0.01 †
	Scrutiny of organ involvement when blood cultures are consecutively positive	33	64.7 (total = 51)	15	57.7 (total = 26)	18	72.0 (total = 25)	0.38 †
(**B**)								
30-day mortality rate		58	28.4	41	34.2	17	20.2	0.04 †
*C. albicans*		90	44.1	56	46.7	34	40.5	0.47 †
*C. parapsilosis*		54	26.5	31	25.8	23	27.4	0.87 †
*C. glabrata*		25	12.3	12	10.0	13	15.5	0.28 †
*C. tropicalis*		17	8.3	9	7.5	8	9.5	0.62 †
*C. guilliermondii*		7	3.4	6	5.0	1	1.2	0.25 †
*C. lustaniae*		4	2.0	1	0.8	3	3.6	0.31 †
*C. krusei*		3	1.5	3	2.5	0	0.0	0.27 †
*C. dubliniensis*		1	0.5	1	0.8	0	0.0	1.00 †
*C. famata*		1	0.5	1	0.8	0	0.0	1.00 †
*C. duobshaemulonii*		1	0.5	0	0.0	1	1.2	0.41 †
*C. metapsilosis*		1	0.5	0	0.0	1	1.2	0.41 †

† Fisher’s exact test; ‡ Student’s *t* test. Abbreviations: ADL, activities of daily living; CRBSI, catheter-related bloodstream infection; IE, Infectious endocarditis; n, number of repeat experiments.

**Table 2:** Relationship between antifungal drugs received by patients and renal function and mortality.

	Echinocandins Group = 106	Nonechinocandin Drugs Group = 85	*p* Value
30-day mortality rate	28.3	27.1	0.87 †
serum creatinine level (median)	0.77	0.99	0.04 ‡

† Fisher’s exact test; ‡ Wilcoxon rank-sum test.

**Table 3:** Logistic regression analysis of the compliance with the actions bundle and 30-day mortality rate.

	Odds Ratio	*p* Value
Collection of two sets of blood cultures	0.88	0.72
Consulting an ophthalmologist	0.52	0.13
Follow-up blood cultures until clearance of candidemia	0.23	<0.01

## Data Availability

Data are contained within the article.

## Abbreviations

IFIs: invasive fungal infections; AST: antimicrobial stewardship team; AFT: antifungal stewardship team; ADL: activities of daily living; CRBSI: catheter-related bloodstream infection; IE: infectious endocarditis; n: number of repeat experiments.
